# Baseline Neuropsychological Characteristics of Adolescents and Young Adults with Down Syndrome Who Participated in Two Clinical Trials of the Drug Memantine

**DOI:** 10.3390/brainsci15111164

**Published:** 2025-10-29

**Authors:** Alberto C. S. Costa, Ana C. Brandão, Veridiana Leiva, H. Gerry Taylor, Mark W. Johnson, Patrícia Salmona, Guilherme Abreu-Silveira, Thomas Scheidemantel, Nancy J. Roizen, Stephen Ruedrich, Richard Boada

**Affiliations:** 1 Department of Psychiatry, Case Western Reserve University, Cleveland, OH 44122, USA; mwj2@case.edu (M.W.J.); thomas.scheidemantel@uhhospitals.org (T.S.); stephen.ruedrich@uhhospitals.org (S.R.); 2 Hospital Israelita Albert Einstein, São Paulo CEP 05652, SP, Brazil; ana.cbrandao@einstein.br; 3 São Paulo Center for Clinical Studies and Research–CEPEC-SP, São Paulo CEP 05615, SP, Brazil; veridianaleiva@yahoo.com.br (V.L.); drapatriciasalmona@drzan.com.br (P.S.); guilherme.pediatra@gmail.com (G.A.-S.); 4 Center for Biobehavioral Health, Nationwide Children’s Hospital Research Institute and The Ohio State University, Columbus, OH 43205, USA; hudson.taylor@nationwidechildrens.org; 5 Department of Pediatrics, Case Western Reserve University, Cleveland, OH 44122, USA; nancy.roizen@uhhospitals.org; 6 Kennedy Krieger Institute, Baltimore, MD 21202, USA; boadar@kennedykrieger.org

**Keywords:** down syndrome, hippocampal deficits, executive function, episodic memory, working memory, neurocognitive phenotype, memantine, NMDA receptor, Alzheimer’s disease

## Abstract

Background/Objectives: Down syndrome (DS) is a neurodevelopmental and neurodegenerative disorder typically caused by trisomy 21. We recently concluded a two-site (Ohio, USA and São Paulo, Brazil), phase-2, randomized trial to evaluate the efficacy, tolerability, and safety of the drug memantine in enhancing cognitive abilities of adolescents and young adults with DS. This trial was a follow-up study to a pilot trial performed in Colorado, USA. Results of these two clinical trials have been published elsewhere. Here, we present a comparative analysis of the baseline neuropsychological assessments at the three sites of these two studies, including their psychometric properties, and an account of the considerations involved in the test battery design. We compared test results in the different sites as a way of evaluating the replicability and generalizability of the test results. The distribution of the test results at each site was analyzed and combined when no differences were detected between the mean values of these results. We used post-treatment data from the placebo arms of these studies to quantify test–retest reliability. Results: Most measures had comparable mean values across test sites, and had good-to-excellent feasibility, few floor effects, and good-to-excellent test–retest reliability. A few measures, however, were deemed unsuitable for use in future studies. Conclusions: This study demonstrated remarkable consistency of results across studies in two countries with significantly different cultures and levels of socioeconomic development, which provides supporting evidence for the future design and implementation of similar multicenter, international clinical studies involving participants with DS.

## 1. Introduction

Down syndrome (DS) is the set of phenotypes of variable expressivity that typically results from trisomy 21 [1,2]. People with DS are especially vulnerable to neurodevelopmental and neurodegenerative disorders. The intellectual disability (ID) displayed by individuals with DS is mostly generalized [3]. However, disproportionate deficits have been observed in neural processes heavily dependent on the hippocampus [4,5], prefrontal cortex [6], and cerebellum [7,8]. Alzheimer’s disease (AD)-type neuropathology is universal by 40 years of age in persons with DS, and the mean age of onset of clinical dementia is approximately 55 years [9,10]. According to recently published criteria [11], all presymptomatic individuals with DS are now classified as having ‘Stage 0’ DS-associated AD (DSAD). Dementia has been reported as the main cause of death of older adults with DS, which makes it a key ‘biological limiter’ for further progress in extending the life expectancy of those with DS [12,13]. Therefore, pharmacological therapies to counteract both the neurodevelopmental and neurodegenerative aspects of DS are major unmet needs.

Based on behavioral and electrophysiological preclinical evidence obtained in mouse models of DS, Costa [14] proposed the hypothesis that dysfunction of the N-Methyl-D-aspartate (NMDA) subtype of glutamate receptors may play significant pathogenic roles in both the neurodevelopmental and neurodegenerative components of DS. A pilot trial of the NMDA receptor antagonist memantine, primarily aimed at testing whether this AD drug could enhance hippocampus-dependent cognitive abilities of young adults with DS, was carried out by some members of our research team more than 10 years ago [15]. Due to its small sample size, this pilot study was expectedly inconclusive. However, post hoc power analysis of the resulting data was encouraging enough to warrant the design and implementation of a phase II, follow-up clinical trial of memantine in adolescents and young adults with DS.

Results of the follow-up memantine trial were recently published [16]. In this work, we showed that 16-week memantine treatment in adolescents and young adults with DS with standard AD dosage of this drug (20 mg/day) was well tolerated, but produced no statistically significant differences between the memantine and placebo arms on the primary measure (California Verbal Learning Test 2nd Edition short-form, CVLT-II-sf, Free-Recall Total Score) or any of the secondary outcome measures (a broad battery of cognitive and adaptive measures). However, because subtherapeutic plasma levels of memantine (i.e., <0.5 μmol/L) were observed in more than 90% of the study participants, we performed a post hoc, exploratory analysis on data from only those in the memantine arm for whom the plasma levels of this drug were at least 0.4 μmol/L. This analysis provided evidence of this drug’s potential effectiveness in improving scores in both the primary measure and one of the secondary measures (forward digit span).

In addition to these two trials, Hanney et al. [17] published results of a randomized clinical trial with memantine in adults with DS 40 years of age or older to test the efficacy of this drug in DSAD. In that study (also known as the MEADOWS study) memantine (10 mg/day) was well tolerated but the treatment produced no significant improvement on either the primary or the secondary efficacy measures in this participant cohort. Given the low dose of memantine used in that study, it is probable that stable therapeutic plasma levels of this drug were never reached in any of the study participants in that trial.

Here, we present an analysis of the baseline neuropsychological data from the follow-up memantine study (i.e., data collected before the beginning of drug intervention) in its two sites (Cleveland, OH, USA and São Paulo, SP, Brazil) side by side with the corresponding data from the pilot memantine study (performed in Denver, CO, USA). The combined dataset from these two studies comprises baseline assessments from 199 unique study participants with DS (67 from Cleveland, 93 from São Paulo, and 39 from Denver). To quantify test–retest reliability for the neuropsychological measures, we used the baseline and post-treatment data from the placebo arms of these studies. By analyzing the statistical and psychometric properties of the baseline test results from each study site and of the combined dataset, we produced a detailed view of the neuropsychological profile of the type of individual with DS likely to be recruited in future studies with similar aims. It is important to emphasize here that the effects of the drug memantine were not considered in this work.

The design and implementation of neuropsychological test batteries in the context of clinical trials with individuals with DS pose several unique and sometimes conflicting challenges. Some of these challenges arise from potential confounders related to cooperation, motivational factors, and attention. In general, measures should be selected based on their construct and content validity across cultures/languages, appropriateness to the level of cognitive functioning of the participant (e.g., no or minimal floor effects), and relevance to core cognitive domains that are disproportionately affected by trisomy 21 (e.g., short-term memory and episodic memory). Acceptable test–retest reliability is also essential. Normally distributed test results (so that parametric statistics can be used) are desirable but not essential. Accordingly, the main goal of this work is to use our dataset to illustrate some of the essential considerations in planning and implementing multisite, multicountry trials designed to test whether pharmacological interventions can enhance cognitive function in individuals with DS. This work should provide useful information to aid the design of similar studies for individuals with DS and may even prove useful in informing and guiding studies involving individuals with other forms of ID.

## 2. Materials and Methods

Study Design. We performed post hoc analyses of baseline, de-identified data from two prospective, double-blind, randomized clinical trials of the drug memantine hydrochloride that were conducted in adolescents and young adults with DS [15,16]. To assess test–retest reliability for neuropsychological measures, we analyzed the post-treatment data from placebo arms of these studies. Both studies used a matched-pair design in which participants were matched by sex and age (within 3 years) and randomly assigned to the memantine or placebo arm of the study (50/50). The studies were conducted ethically, in accordance with the World Medical Association Declaration of Helsinki. The original protocol for the pilot memantine study was approved by the Colorado Multiple Institutional Review Board, University of Colorado Anschutz Medical Campus, CO, USA (#06-0934; ClinicalTrials.gov identifier: NCT01112683). The follow-up memantine trial was approved by University Hospitals’ Institutional Review Board, Cleveland Medical Center, OH, USA (#06-14-41; ClinicalTrials.gov identifier: NCT02304302). The São Paulo site of this follow-up study also received approval by the Hospital Israelita Albert Einstein’s IRB (#1.543.943) and the Brazilian Federal Ethics Committee (CONEP, CAAE: 54952916.7.0000.0071).

Study Participants. In the two clinical trials, persons with DS of both biological sexes were recruited in conjunction with regional and national DS associations and local and regional DS clinics. Eligibility ages for the pilot memantine study were 18 to 32 years. For the follow-up study, these ages were 15 to 32 years. Given that both trials were designed by the same two investigators (A.C.S.C. and R.B.), these studies share the same set of inclusion and exclusion criteria, which are listed in Appendix A. In brief, we aimed to recruit individuals with cytogenetically documented trisomy 21, or complete unbalanced translocation of chromosome 21, and in general good health. A reliable family member or caregiver agreed to accompany the participant to all visits, provide information about them as required by the protocol, ensure compliance with the medication schedule, and help them complete reliably the study assessments. There was no specific cognitive level used to exclude participants. Instead, the principal investigator of each site, based on his/her experience, made a clinical determination regarding each participant’s ability to cope with the demands of the study in consultation with parents/caregivers.

Study Procedures. For a complete description of the experimental methods used in the pilot memantine trial, we direct the reader to Boada, Hutaff-Lee, Schrader, Weitzenkamp, Benke, Goldson and Costa [15] and the available information at ClinicalTrials.gov (NCT01112683). The procedures used in the follow-up memantine trial can be found in Costa, Brandao, Boada, Barrionuevo, Taylor, Roth, Stasko, Johnson, Assir, Roberto, Salmona, Abreu-Silveira, Bederman, Prendergast, Huls, Abrishamcar, Mustacchi, Scheidemantel, Roizen and Ruedrich [16] and at ClinicalTrials.gov (NCT02304302). Briefly, for both studies, the engagement with the participant’s families always started with an initial telephone screen to assess probable eligibility. This was followed by a 2-h-long screening visit in which the research objectives and the design of the clinical trial were explained thoroughly, and the informed consent and assent were obtained. After this visit, we scheduled a formal baseline medical appointment, involving a 1-h visit in which a complete clinical history was collected, and physical examination and clinical laboratory evaluations were performed. Neuropsychological assessments occurred in a third, standalone 2–2.5-h visit (T1), in which tests were applied methodically and interactively, with several minutes of resting time between different tests. Study medication (memantine or placebo, randomly assigned to each participant) was dispensed at the end of this visit. Participants returned for follow-up medical evaluations at 8 and 16 weeks later. The final neuropsychological assessment also occurred 16 weeks after the beginning of the study medication dispensation (T2).

Neuropsychological Test Battery. In both clinical studies, the neuropsychological battery was divided into four main domains: (1) ‘hippocampal measures’ (requiring explicit long-term memory); (2) ‘prefrontal measures’ (requiring short-term/working memory and executive function); (3) language measures; and (4) cognitive and adaptive functioning.

The primary hypothesis for both the pilot and the follow-up trials was that the drug memantine was expected to improve scores in hippocampal measures. For the pilot trial, the primary efficacy measures consisted of the Pattern Recognition Memory (PRM) and the Paired-Associate Learning (PAL) subtests of the Cambridge Neuropsychological Test Automated Battery (CANTAB) administered at the baseline session and a second testing session at 16 weeks of medication treatment. The choice of these two measures was based on the availability of published mean and standard deviation values for these tests in individuals with DS [4], which allowed us to make power calculations of minimum required sample sizes. In addition to these primary measures, two secondary measures sensitive to hippocampus function were also administered: the CVLT-II-sf and the Rivermead Behavioral Memory Test-Children’s version (RBMT). Additional measures were selected primarily to characterize the participant sample. These included two prefrontal-cortex-dependent measures: CANTAB Spatial Working Memory (SWM) and Recall of Digits Forward from the Differential Ability Scales-Second Edition (DAS-II). In addition, we applied two measures to assess language functioning: Peabody Picture Vocabulary Test-3rd edition (PPVT-III); Test of Reception of Grammar-2nd edition (TROG-II); and the DAS-II Verbal Fluency test. Finally, we used the DAS II Matrices and the Scales of Independent Behavior–Revised (SIB-R) as measures of overall cognition and adaptive functioning.

Because the CVLT-II-sf was the only test in which we were able to detect a significant drug effect in the Denver pilot trial, this test became the primary measure for the Cleveland/São Paulo, follow-up memantine study. Accordingly, we hypothesized that the participants in the memantine arm of the trial would show a greater improvement from baseline to the 16-week visit than the placebo group on this measure of episodic memory. Our experience in conducting the pilot trial also informed the choices of the secondary neuropsychological measures assessed in the follow-up memantine trial. We also made the decision to keep measures of receptive semantics and grammatical understanding in the test battery, the PPVT; TROG-II; and the DAS-II Matrices. These measures were predicted to remain relatively stable, thus acting as benchmarks against which to compare any potential improvements in long-term memory or short-term/working memory.

An additional secondary hypothesis was that memantine might decrease the frequency or severity of behavioral issues (although we found no indication of this potential effect in the pilot trial). To test this specific hypothesis, the SIB-R parent-filled questionnaire was also administered. A final design consideration was that, similar to the pilot study, we had to be able to apply this new test battery in a single session and within no more than 2.5 h.

Translation of Neuropsychological Tests Not Available in Brazilian Portuguese. A major hurdle for the implementation of the follow-up memantine clinical trial in Brazil was that many of the tests used in Denver for the pilot memantine trial were neither translated nor validated in Portuguese. This was not a significant issue for the computer-based tests of the CANTAB (PRM, PAL, SWM, Spatial Span or SSP). Because these tests present visual patterns on the computer screen and place few demands on language comprehension, only a simple translation of the ‘Instructions to Participants’ was needed to administer them in Portuguese. The same was the case for the Go/No-go test and the DAS II Recall of Digits and Matrices. Although the translation of the PPVT-III and TROG-II was straightforward, special care had to be taken in terms of choosing high-frequency Portuguese words and maintaining comparable degrees of grammatical complexity for the questions related to the picture choices in the TROG-II. It was precisely the CVLT-II-sf, however, that required the most work. The CVLT-II-sf, the primary measure for the follow-up memantine trial, is a word list test that is read to the examinee with no pictorial aid. For this test, team members who were fluent in both English and Portuguese selected words of comparable meaning. Table A1 contains the original CVLT-II-sf English word list and its Portuguese counterpart.

Statistical Analyses. We compared the sample means for test results obtained in two or three different sites as a way of evaluating their replicability and generalizability. For this analysis, we used Statistica Academic Software version 13 (TIBCO Software, Palo Alto, CA, USA) to generate descriptive statistics and perform Student’s *t*-tests with Welch’s correction (when comparing results from the two sites in the follow-up study) or one-way Analysis of Variance (ANOVA) with Bonferroni post hoc tests (when comparing the means of the test results from all three sites across the two studies). In this analysis, each neuropsychological measure was treated as an independent assessment, with no adjustment for multiple testing, which can be justified when research outcomes are treated as exploratory results, which require further confirmatory studies to support them [18]. Statistica was also used to calculate Pearson’s r correlations, perform principal component analysis (PCA), and implement multiple linear regression analyses. Intra-class correlations (ICC) for all measures, except for SIB-R, were computed using a 2-way mixed effects model [19], single measurement and absolute agreement in IBM SPSS 28 (SPSS, Chicago, IL, USA). For the SIB-R Broad Independence measure, a 2-way random effects model was used to account for parent raters. We used the Prism software version 7 (GraphPad Software Inc., San Diego, CA, USA) to generate histogram representations of the frequency distributions of the test results and to produce nonlinear regression fits of the data according to unimodal Gaussians (Y = Amplitude × exp{−0.5 × [(x − $\bar{x}$)/s]^2^}); which included Shapiro–Wilk normality test), single exponential decay (Y = (Y0 − Plateau) × exp(−x/τ) + Plateau; which included an extra sum-of-square F test to compare the time constant “τ” between scores distributions between sites), or second-order polynomial (Y = B0 + B1 × x +B2 × x^2^) functions. Two-tailed tests at a type I error rate of 5% were used. Results of these analyses were represented graphically as mean ± 95 confidence interval (CI).

## 3. Results

### 3.1. Demographics

**Figure 1:**
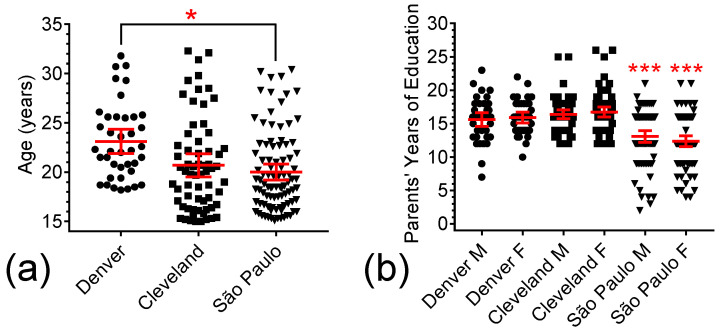
Ages and SES of Participants in each study site. (**a**) Scatter plot of participants’ age in the Denver (●), Cleveland (■), and São Paulo (▼) sites. (**b**) Scatter plot of participants’ SES in the Denver, Cleveland, and São Paulo sites, as assessed by years of education of their mothers (M) and fathers (F). Red lines represent means ± 95% CI; “*” represents *p* < 0.05 and “***” *p* < 0.001.

The age range for study participation in the pilot memantine trial was 18 to 32 years, whereas it was 15 to 32 years in the follow-up memantine study. In Figure 1a, we illustrate the individual age in years at baseline for the study participants of the pilot memantine study in Denver, and the Cleveland and São Paulo sites of the follow-up memantine study. Given the inclusion of participants younger than 18 years of age in the follow-up memantine study, it is not surprising that ANOVA revealed a significant difference in the mean age among the groups (F(2, 196) = 4.252, *p* = 0.0156). Bonferroni post hoc tests showed that the participants in the Denver site were significantly older (mean age = 22.4 years) than those from São Paulo (mean age = 20.0 years; *p* = 0.0120), but not significantly older than those from Cleveland (mean age = 20.8 years; *p* = 0.1982). Additionally, no significant differences were detected in mean ages between the Cleveland and São Paulo sites (*p* = 0.7608).

The number of years of formal school education for the participant’s parents was used as a proxy for socioeconomic status (SES). The values of this measure for each site cohort are illustrated in Figure 1b. Once again, we found that site location had a significant effect on group means (F(2, 369) = 52.693, *p* < 0.0001). The mean number of years of education of the mothers and fathers from Denver (15.9 and 15.6 years, respectively) were statistically comparable to those from Cleveland (16.4 years; *p* = 1.0000, and 16.7 years; *p* = 1.0000). In contrast, the SES of both participants from Denver and those from Cleveland was significantly higher than for those from São Paulo, as quantified by the mean number of years of mother education (13.1 years; *p* = 0.0003 and *p* < 0.0001; respectively) and years of father education (12.4 years; *p* < 0.0001 and *p* < 0.0001, respectively).

### 3.2. Episodic Verbal Long-Term Memory and Working/Short-Term Memory as Assessed by the CVLT-II-sf and the DAS-II Recall of Digits Forward

The CVLT-II-sf is a broadly applied and well-validated instrument in clinical and experimental neuropsychology that is used to assess supraspan word learning ability as a proxy of episodic memory capacity [20]. In the standard CVLT-II, 16 nouns are read in loud voice to the participants. In its short form (sf), which is designed for those with more severe cognitive deficits, it only uses nine words. The CVLT-II-sf was used as a secondary measure in the Denver pilot trial [15] and as the primary measure in the memantine follow-up trial [16]. CVLT-II performance is generally assessed by the total number of target items correctly recalled, summed across four learning trials (Free-Recall Total) and total Free-Recall Discriminability for the learning trials, which considers words recalled correctly as well as words reported that were not on the list, referred to as intrusions.

**Figure 2:**
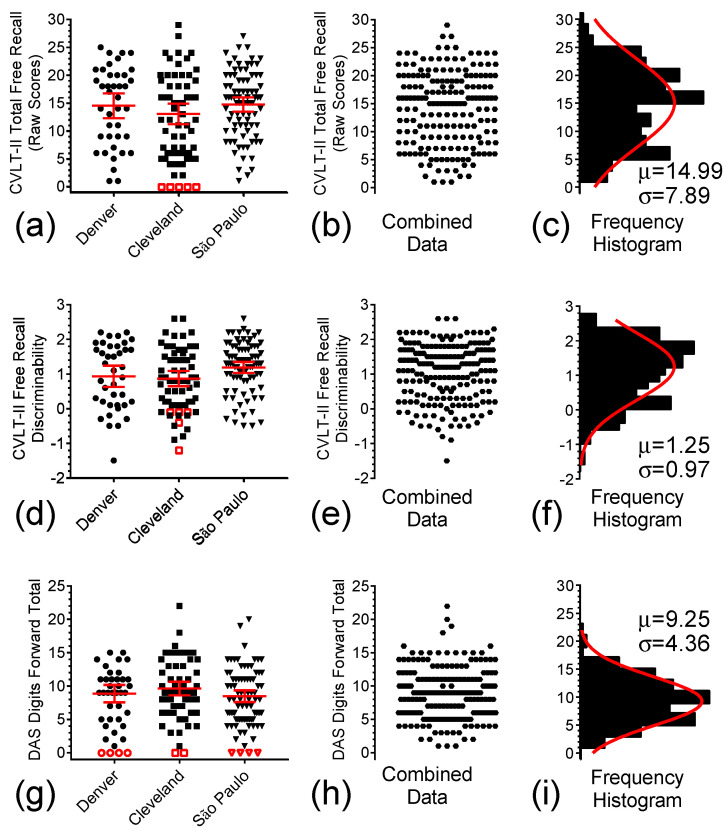
Distribution of individual baseline test scores for the two sub-measures of the CVLT-II-sf and the DAS-II Digits Forward from the pilot memantine study in Denver (●), and from the Cleveland (■), and São Paulo (▼) sites for the follow-up memantine study. (**a**) Scatter plots of Total Recall scores in Denver, Cleveland, and São Paulo (n = 85). (**b**) Combined Total Recall scores for the three study sites. (**c**) Frequency histogram and single Gaussian curve fitting for data depicted in (**b**). (**d**) Scatter plots of Total Recall Discriminability scores in Denver, Cleveland, and São Paulo. (**e**) Combined scores for the three study sites. (**f**) Frequency histogram and single Gaussian curve fitting for the combined data. (**g**,**d**) Scatter plots of the DAS-II Digits Forward scores, (**h**) Combined scores. (**i**) Frequency histogram and single Gaussian curve fitting for the combined data. Solid symbols represent the computed data, open symbols are the excluded floor scores, and middle solid lines and error bars in the graphs represent mean ± 95% confidence interval (CI).

Figure 2a–f depicts the individual Free-Recall Total raw scores and Free-Recall Discriminability scores for the participants in the three study sites. (In this and all similar graphs in this paper, open symbols represent floor scores, which were not used in statistical comparisons between groups.) ANOVA of the data in Figure 2a did not reveal any significant difference in the mean Free-Recall Total raw scores among the three study sites (See Table 1 for statistical results). Consequently, in Figure 2b, the data of these sites were combined, which generated the histogram shown in Figure 2c. The non-linear curve fitting of the frequency histogram resulting from these data produced a Gaussian curve with a mean sample value ($\bar{x}$) of 14.99 (95% CI 12.67 to 17.15) and standard deviation (s) of 7.89. In panels d-f of Figure 2, a similar analysis was performed for the Free-Recall Discriminability scores. (Note that, for consistency, the open symbols here refer to the data from the same individuals with floor performance as assessed by the Free-Recall Total raw scores.) Although the *p* value was very close to reaching significance criterion, no significant difference was found for the mean Free-Recall Discriminability scores among the three study sites. Therefore, data were combined in Figure 2e, and these combined data were fit with a Gaussian function with $\bar{x}$ = 1.26 (95% CI 1.04 to 1.49) and s = 0.97 (95% CI 0.76 to 1.27).

Significant deficits in verbal short-term memory/working memory in individuals with DS have been well documented and have been detected even in comparisons made with persons with ID of other etiologies matched by vocabulary knowledge [reviewed in Basten, Boada, Taylor, Koenig, Barrionuevo, Brandao and Costa [21]]. In the present work, Figure 2g represents individual DAS-II Recall of Digits [22] raw scores as a measure of forward verbal digit span for the participants in the three study sites. ANOVA of these data did not reveal any location effect for forward digit span among the participants in the three study sites (Table 1). Therefore, we pooled the data from these three sites, as shown in Figure 2h, to generate the frequency histogram and single Gaussian curve fitting shown in Figure 2i.

### 3.3. CANTAB PRM, PAL, and SWM

**Figure 3:**
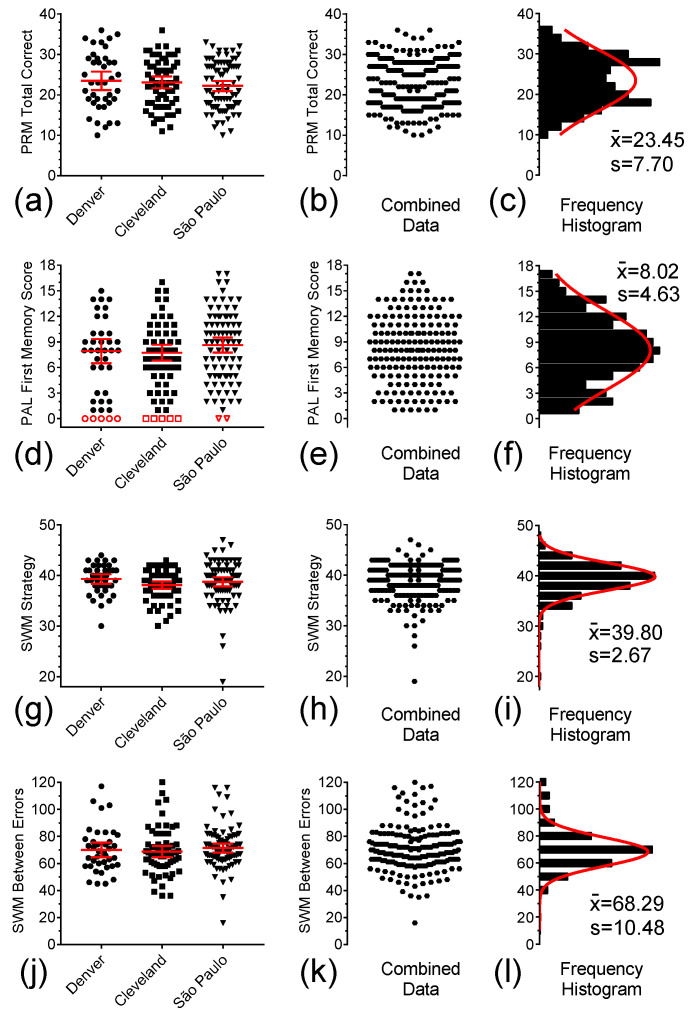
Distribution of individual baseline test scores for the CANTAB PRM, CANTAB PAL, and CANTAB SWM Strategy and Between Errors in Denver (●), and from the Cleveland (■), and São Paulo (▼) sites. (**a**,**d**,**g**,**j**) Scatter plots of these tests scores, respectively. (**b**,**e**,**h**,**k**) Combined scores for each of these four measures at the three study sites. (**c**,**f**,**i**,**l**) Frequency histograms for combined scores and single Gaussian curve fittings of the combined data for each of the four measures in Denver, Cleveland, and São Paulo. Solid symbols represent the computed data, open symbols are the excluded floor scores, and middle solid lines and error bars in the graphs represent mean ± 95% CI.

**Figure A1:**
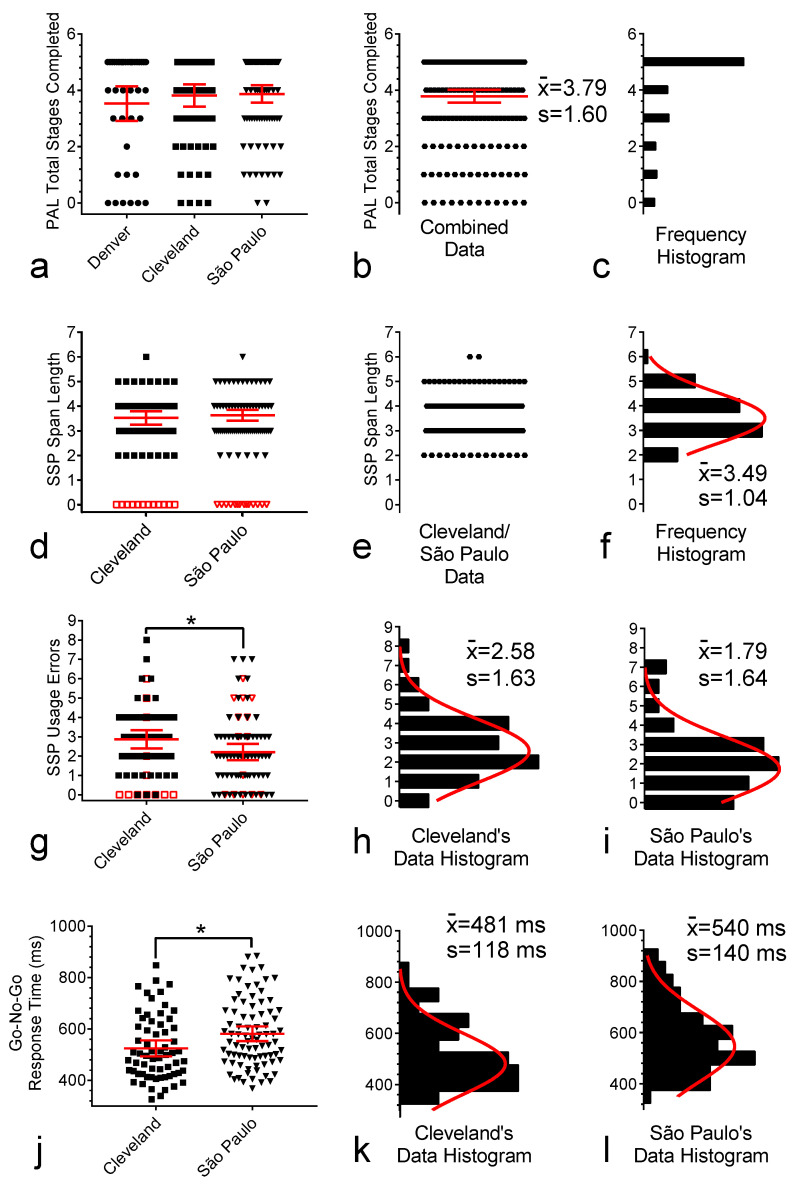
Distribution of individual baseline PAL Stages Completed, SSP Spam Lengths and Usage Errors, and Go-No-Go test scores in Denver (●), Cleveland (■), and São Paulo (▼). (**a**,**d**,**g**,**j**) Scatter plots of scores in each of the three or two sites. (**b**,**e**,**h**,**k**) Combined test scores for each of these four measures at either three or two study sites. (**c**,**f**,**i**,**l**) Frequency histograms for combined scores and single Gaussian curve fittings of the combined data for each of the four measures in Denver, Cleveland, and São Paulo. Solid symbols represent the computed data, and open symbols are the excluded floor scores; red lines in (**a**,**d**,**g**,**j**) represent mean ± 95% CI; “*” represents *p* < 0.05.

The PRM and PAL of the CANTAB [23,24] are measures of long-term visual recognition memory and visual episodic memory, respectively. There is extensive evidence demonstrating that individuals with lesions of the temporal lobe display marked impairment on measures of PAL [25]. Pennington, Moon, Edgin, Stedron and Nadel [4] were the first to observe that participants with DS had greater difficulty recognizing a previously presented pattern on the PRM and scored more poorly on the PAL when compared to mental-age control participants. In Figure 3a,d one can observe the values of PRM Total Correct Choices and the PAL First Memory Scores, respectively, for participants in the three sites. ANOVA did not reveal location effects for either (Table 1). Consequently, we pooled test-specific data from these three sites, as shown in Figure 3b,e, to generate the frequency histograms and single Gaussian curve fittings depicted in Figure 3f,i. PAL Stages Completed sub-measure results was clearly not distributed normally and are illustrated in Figure A1a–c.

The CANTAB SWM is a convenient way to assess spatial working memory [26]. This task has been used in clinical trials and has been deemed an appropriate test in participants with DS [6]. Figure 3g,j depict the individual SWM Strategy and Between Errors subscores for the participants in the three study sites. No location effect was detected for this measure among the three study sites (Table 1), which led to the pooling of the data from the three sites into the graphs shown in Figure 3h,k and the frequency histograms and single Gaussian curve fittings shown in Figure 3i,l.

### 3.4. SSP and Go-No-Go

Because we had observed a trend toward significance for the Recall of Digits Forward from the DAS-II in the pilot study, this test was also chosen to be part of the new trial, along with two additional prefrontal cortex-dependent tasks: CANTAB SSP and a simple Go/No-go test [27]. Test results for these measures are summarized in Figure A1d–l and Table 1.

### 3.5. DAS-II Matrices, PPVT, TROG-II, and SIB-R

**Figure 4:**
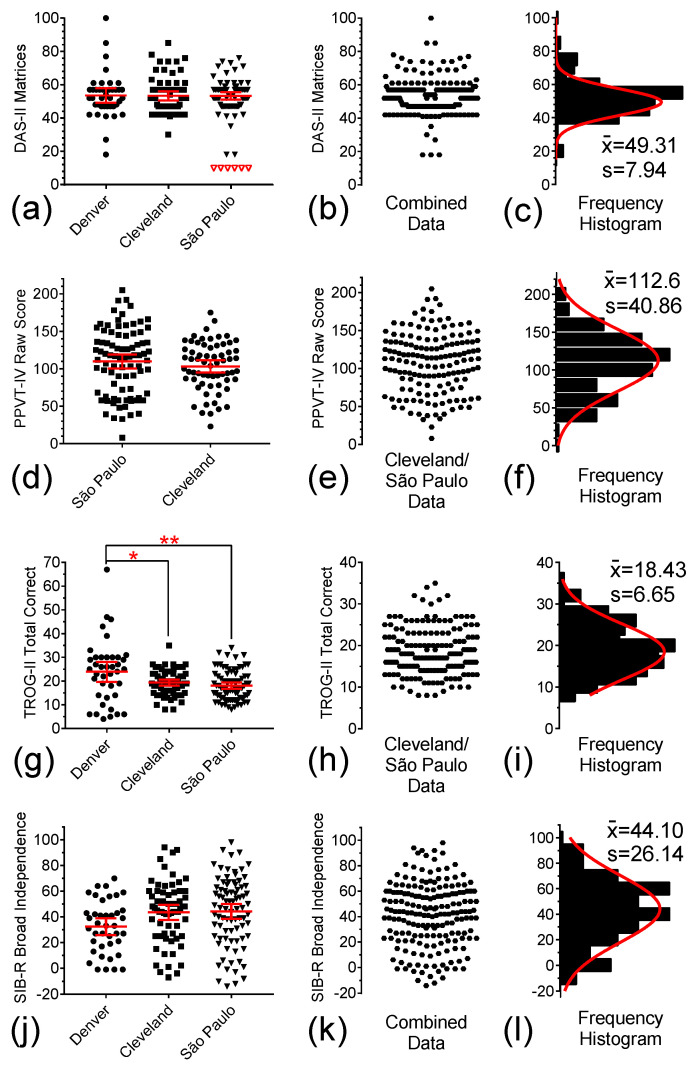
Distribution of individual baseline test scores for DAS-II Matrices, PPVT-IV, TROG-II, and SIB-R Broad Independence in Denver (●), and from the Cleveland (■), and São Paulo (▼) sites. (**a**,**d**,**g**,**j**) Scatter plots of DAS-II Matrices scores, CANTAB PRM Total Correct answers, TROG-II Total Correct choices, and SIB-R Broad Independence scores, respectively. (**b**,**e**,**h**,**k**) Combined scores for each of these four measures at the three study sites. (**c**,**f**,**i**,**l**) Frequency histograms for combined scores and single Gaussian curve fittings of the combined data for each of the four measures in Denver, Cleveland, and São Paulo. Solid symbols represent the computed data, open symbols are the excluded floor scores, and middle solid lines and error bars in the graphs represent mean ± 95% CI. The symbols “*” and “**” indicate “*p*” values < 0.05 and <0.01.

Figure 4 show the distributions of four benchmark ability measures in our study population, which were used to assess intellectual functioning and adaptive behavior as potential secondary tolerability measures in both the pilot [15] and follow-up [16] memantine studies.

The DAS-II Matrices was used as a measure of non-verbal reasoning ability [22]. Because age norms are not available for individuals older than 17y11m, the ‘ability score’ was used as the dependent variable. Figure 4a depicts individual DAS-II scores for the three study sites. No location effect was detected. Accordingly, pooled data are shown in Figure 3b and the resulting frequency histogram with a single Gaussian curve fitting is shown in Figure 3c.

The PPVT was used to assess one-word receptive language skills [28]. The 3rd version of this test was used in the pilot memantine trial and its 4th version in the follow-up trial. Therefore, to avoid test-version-dependent variabilities, only data from the follow-up memantine trial was analyzed here. Figure 4d represents individual baseline PPVT raw scores at the Cleveland and São Paulo study sites. There was no location effect detected. Data pooled from these two sites are shown in Figure 4e. The resulting frequency histogram and Gaussian curve fitting are shown in Figure 4f.

The TROG-II is a measure of receptive syntax skills with a 0.85 correlation with composite measures of Verbal IQ from the Wechsler Intelligence Scale series [29]. Figure 4g depicts individual TROG-II scores for the participants in the three study sites. Because ANOVA of these data showed a significant location effect (F(2, 187) = 6.501, *p* = 0.0019), we performed Bonferroni tests to identify the source of the location effect. We found out that the mean value of the test results at the Denver site was significantly higher than both the Cleveland (*p* = 0.0206) and São Paulo (*p* = 0.0014) sites, but no significant difference was detected between Cleveland and São Paulo (*p* = 1.000). Although the high mean of the Denver results seems to be driven primarily by a few outliers, we only combined data from the Cleveland and São Paulo in our analysis, which is shown in Figure 4h. The resulting frequency histogram and single Gaussian curve fitting are shown in Figure 4i.

The SIB-R is considered an adequate tool for assessing adaptive functioning domains. It has a wide age range, good psychometric properties, and a history of being used in intervention trials in participants with DS [15]. Figure 4j depicts the distribution of individual SIB-R scores for the participants in the three study sites. Although ANOVA of these data hinted at location dependence (F(2, 185) = 3.058, *p* = 0.049), post hoc analysis failed to confirm any significant differences between the means of any two separate locations (Denver vs. Cleveland, *p* = 0.1159; Denver vs. São Paulo, *p* = 0.0565; Cleveland vs. São Paulo, *p* = 1.0000). Accordingly, data from these three sites were pooled, as shown in Figure 4k, and the resulting frequency histogram with a single Gaussian curve fitting is shown in Figure 4l.

### 3.6. Test Battery’s Psychometric Landscape

#### 3.6.1. Additional Descriptive Statistics

Table A2 provides additional information on the statistical properties of the baseline score histograms that could be fitted by a unimodal Gaussian distribution. This table included number of participants (minus participants at floor), percentage of participants at absolute floor, the arithmetic means and standard deviations, medians, minimum and maximum values, low and upper quartiles, skewness, and kurtosis.

**Figure A3:**
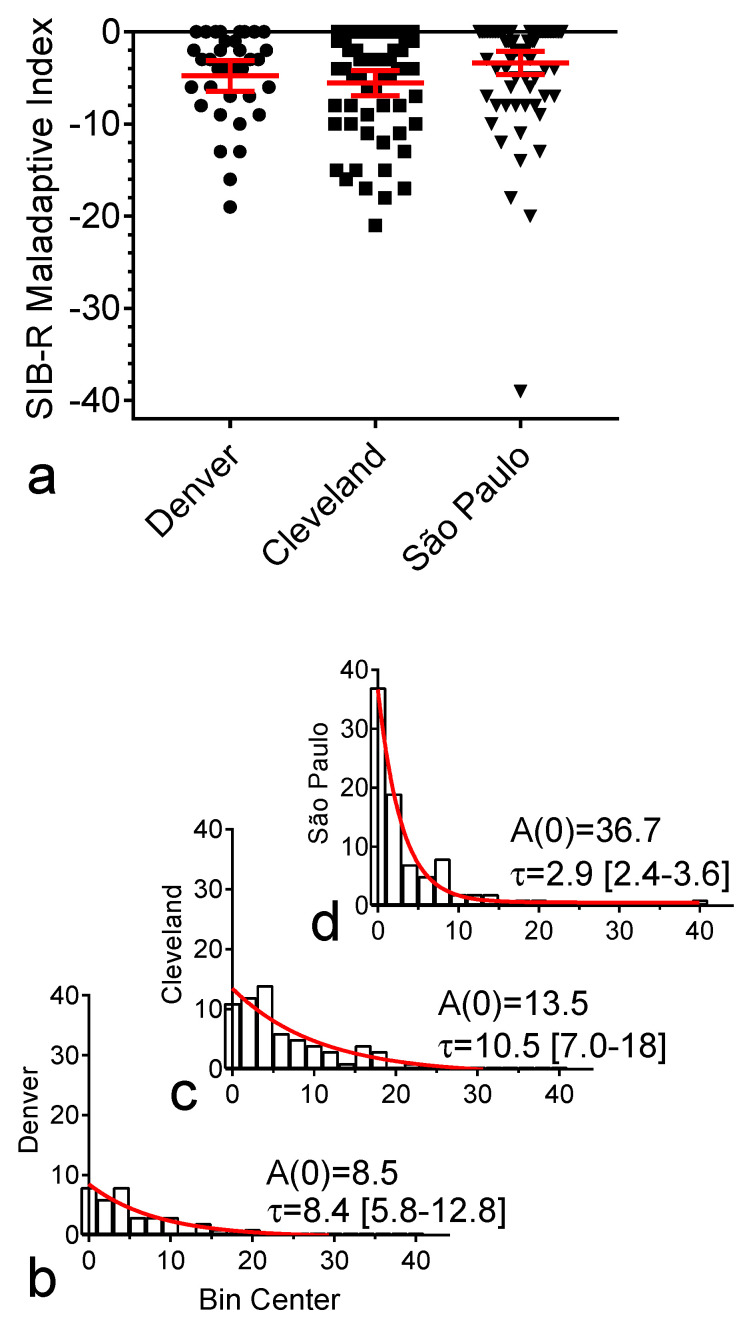
Distribution of individual baseline test scores for the SIB-R Maladaptive Index in Denver (●), Cleveland (■), and São Paulo (▼). (**a**) Scatter plots of test scores in the three sites. (**b**–**d**) Frequency histograms for scores in each of the three sites and single exponential curve fittings of the data in Denver, Cleveland, and São Paulo. Exponential time constants (τ) are values depicted in this figure represent Mean [95% CI]. R^2^ values for the curve fits were 0.89; 0.88; and 0.97; for the Denver, Cleveland, and São Paulo sites, respectively. Extra sum-of-squares F test rejected the null hypothesis that all distribution histograms could be best fitted by exponentials with the same “τ” value (F(2, 54) = 28.67, *p* < 0.001).

As illustrated in the previous sections, the histogram of the combined test results for most measures could be fitted with a unimodal Gaussian distribution. However, the PAL total stages completed (see Figure A1) and SIB-R maladaptive scores (Figure A3) were clearly not normally distributed, which is why these sub-measures were not included in Table 1. Most measures were approximately symmetric in their distribution, with only the SSP Usage Errors and Go-No-Go being moderately skewed, and the two SWM sub-measures (Strategy and Between Errors) being heavily skewed. Finally, the distribution of most measures can be considered mesokurtic, except for the two SWM sub-measures and the DAS-II Matrices Ability Scores, which were found to be leptokurtic.

#### 3.6.2. Multiple Regression Analysis

A general linear regression model (Statistica) was used to assess the potential relationship between site location, sex, age, and mother and father’s years of education and the participants’ performance in all 14 measures listed in Table 1. This analysis confirmed the absence of any statistically significant effects of sex (F(14, 81) = 1.2250, *p* = 0.2740; Wilks test), age (F(14, 81) = 1.4486, *p* = 0.1504), mother’s years of education (F(14, 81) = 1.1895, *p* = 0.2994), and father’s years of education (F(14, 81) = 1.8130, *p* = 0.0505) on the test results. However, in contrast to the ANOVA results, a small but significant effect of the test location (site) was identified with this general linear regression approach (F(14, 81) = 1.8319, *p* = 0.0475), with post hoc significant effects detected for the CVLT-II-sf Total Score (*p* = 0.0120) and Free-Recall Discriminability (*p* = 0.0028), SSP Span Length (*p* = 0.0039), and PPVT-4 (*p* = 0.0063).

#### 3.6.3. Test–Retest Reliability

We used baseline (T1) and post-treatment (T2) aggregate data from the placebo arms at the three sites to assess test–retest reliability for various measures. (Aggregate T2 and T1 for only two of three sites were used when either the mean tests scores were statistically different in one of the sites or when test results were only available for the Cleveland and São Paulo sites.) The results of this analysis are shown in Table 2 and includes mean ± SD values for T1 and T2, mean ± SD values for the difference between T2 and T1, Cohen’s “d” and ICC values.

All but 4 of the 15 measures had Cohen’s d’s below 0.2. The four exceptions were the two sub-measures of the CVLT-II-sf (Cohen’s d = 0.447 and 0.485, for Free Recall Total and the Free-Recall Discriminability scores, respectively), the PAL 1st Memory Score (Cohen’s d = 0.206), and the SIB-R Broad Independence score (Cohen’s d = 0.231).

In terms of ICC values, one measure out of the 15 analyzed (PPVT-4 Raw Score) had “excellent” test–retest reliability (ICC > 0.90); 8 measures had “good” reliability (0.90 > ICC > 0.75); 5 measures were in the “moderate” range (0.75 > ICC 0.50), and only one measure (SSP Usage Errors) had an ICC in the poor range (<0.50).

#### 3.6.4. Potential Functional Clustering of the Different Measures

We calculated Pearson’s “r” correlations between the different measures comprising the test battery to examine potential relations among them. The results of this analysis are summarized in Table A3. Although “*p*” values indicated significant correlation (<0.05; indicated by asterisks) between several measures, only 17 “r” values ≥ 0.5 were found; which were denoted by boldface digits in Table A3. Among these were the expected correlations between sub-measures of the same test, such as the CVLT-II-sf Free Recall Total and the Free-Recall Discriminability scores and PAL 1st Memory Score and Total Stages Completed. Similarly, there was an expected inverse correlation between the SMW Strategy and Between Errors. Correlations with “r” values ≥ 0.5 were also observed between PPVT-4 Raw Scores and CVLT-II-sf Free Recall Total, CVLT-II-sf Free-Recall Discriminability, DAS Recall of Digits, PRM Total, SSP Span Length, TROG, and SIB-R scores. The DAS Recall of Digits also showed ‘appreciable’ direct correlation with the CVLT-II-sf Free-Recall Discriminability scores, SSP Span Length, and TROG, whereas an inverse correlation was found between this measure and SMW Between Errors.

**Figure 5:**
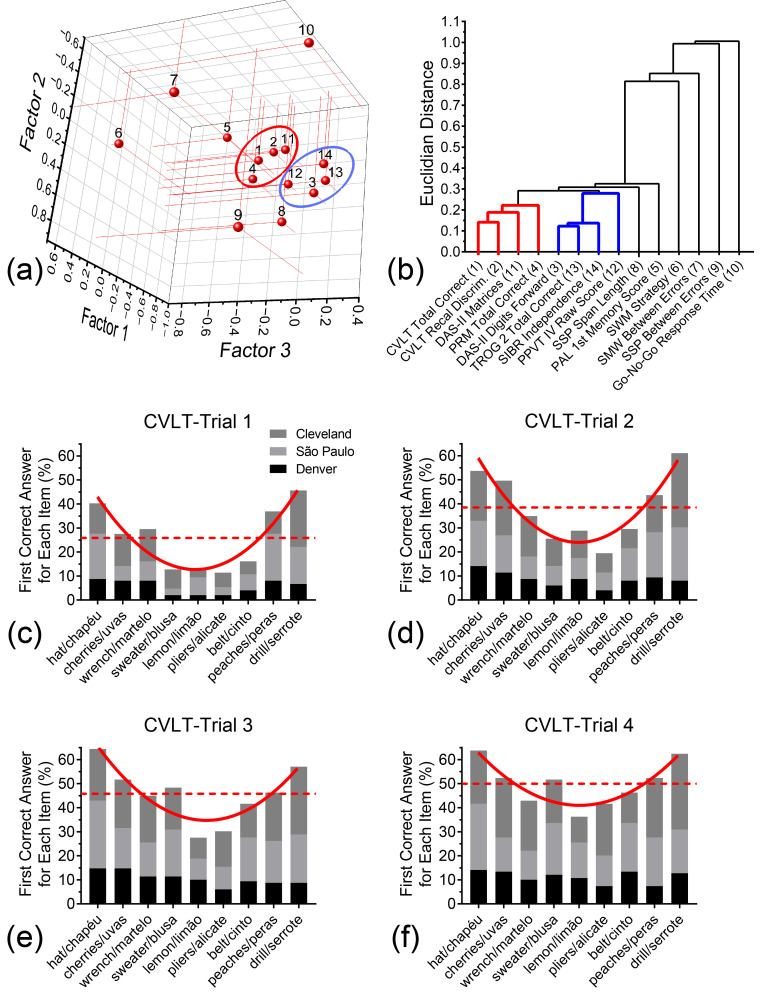
Principal components and hierarchical clustering analyses of test battery’s measures and CVLT-II-sf distribution of responses at baseline in Denver, Cleveland, and São Paulo. (**a**) 3-D scatter plot representation of the three principal factor coordinates calculated for the 14 core measures of the test battery. (**b**) Hierarchical clustering of the measures based on the Euclidian distance between variables. Ovals in 3-D plot and thick lines in the dendrogram represent two emergent cognitive domain clusters. (**c**–**f**) Bar graph representations of successful memory recall for words as a function of their position in the CVLT-II-sf word list in trials 1–4, respectively. The U-shaped curves are second-order polynomial curve fit of the combined CVLT-II-sf serial position distribution of study participants in Denver, Cleveland, and São Paulo.

To further explore potential functional grouping of these different measures, we performed principal component analysis (PCA) to calculate the Euclidian distances between the measures as described by their first three principal components, or factors. These three factors contributed to 57.38% of the total variance (Factor 1 = 38.39%; Factor 2 = 9.90%; Factor 3 = 9.09%). The complete listing of these distances (and loading magnitudes for each measure) can be found in Table A4, and are depicted graphically in Figure 5a,b, which indicate two main clusters. The first cluster comprises the CVLT-II-sf Free Recall Total, CVLT-II-sf Free-Recall Discriminability, DAS Matrices, and PRM Total scores. The second cluster includes the DAS Recall of Digits, TROG, SIB-R, and PPVT-4 scores. It is worth noticing that small Euclidian distance separates these two ‘functional clusters’ from each other, which may reflect an interaction between working memory and long-term memory [30] as assessed by the instruments used in this test battery.

#### 3.6.5. Primacy and Recency Effects for the CVLT-II-sf

Primacy and recency effects are thought to represent an interaction in the recruitment of working memory and long term-memory systems required to recall supraspan word lists [31,32,33]. Evidence for such effects would suggest that participants are indeed making use of both working memory and longer-term storage to recall these word lists. Figure 4c–f show percentage counts for the first correct answer of each item according to their position in the list (1–9) for all three sites across trials 1–4, respectively. (Counts excluded repeated answers and intrusions.) In trial 1 [Figure 5c], recency effects were observed for CLVT-II sf list words in positions 8 and 9, whereas primacy effects were also observed for list words 1, 2, and 3. Similar to what has been reported for the Memory Assessment Scales (MAS) List [34], the typical U-shaped serial position curve diminishes with repeated exposure to the word list [Figure 5c–f].

## 4. Discussion

In the present study, we described the neuropsychological profile of adolescents and young adults with DS who participated in two clinical trials of the drug memantine. This analysis of the baseline neuropsychological data from the follow-up memantine study in two study sites (Cleveland and São Paulo), combined with corresponding data from the pilot memantine study (Denver), provides a rich database of assessments of individuals with DS who fit the typical neuropsychological profile of participants likely to be recruited in future studies with similar aims. This also means that this analysis should not be considered representative of the entire population of individuals with DS living in the community. Overall, we found that most measures in the test battery used in these two memantine trials had comparable mean values across test sites, and had good-to-excellent feasibility, few floor effects, and good-to-excellent test–retest reliability. Additionally, the empirical distribution of the data for most measures was bell-shaped and could be fitted with a single Gaussian curve.

Several neuropsychological test batteries have been developed or adapted for use in research involving adolescents and young adults with DS [21]. Commonly used batteries include the Arizona Cognitive Test Battery (ACTB), which was designed for individuals with DS aged 7–38 years, with an emphasis on low floor effects and targeting hippocampal, prefrontal, and cerebellar systems [6]. Performance-based batteries derived from the ACTB have been used in the ‘Down Syndrome Cognition Project’, which has included participants with DS aged 6–25 and has demonstrated reasonably strong psychometric properties and fidelity of administration across multiple research sites [35]. The TESDAD battery offers a complementary framework that emphasizes direct assessment of mnemonic and executive components [36] and has been employed in a clinical trial [37]. The NIH Toolbox has also been adapted for use with individuals with DS [38], offering standardized assessments of attention, processing speed, and working memory. In addition, the Leiter International Performance Scale-Revised (Leiter-R) and the Kaufman Assessment Battery for Children (KABC) are often employed due to their nonverbal components, making them more accessible for individuals with language impairments, including those with DS [39,40].

As mentioned in Introduction, there are many hurdles to the rigorous design and implementation of neuropsychological test batteries to be used in clinical trials with individuals with DS. Measures should be selected based on their sensitivity, specificity, and relevance to core cognitive domains that are disproportionately affected by trisomy 21. Psychometric properties, such as acceptable test–retest reliability and minimum floor effects, are also essential. Other factors such as duration of test administration and potential cognitive interference between tests being administered consecutively should be considered. Also ideally, battery instruments with a verbal component to be used on different international sites should be available in translated and validated versions in all local languages. Finally, it is critical that the hypothesis driving the design of the trial be taken into account. For example, the two memantine clinical trials from which we extract the baseline neuropsychological data used in the present study were designed to investigate potential cognitive effects of short-term administration of memantine in adolescents and young adults with DS. Therefore, measures assessing adaptive behavior and general intelligence were considered only as descriptive/benchmark measures. Additionally, no quality-of-life measures were included in the battery. If instead we had been aiming to investigate the effect of long-term use of memantine on neurodegeneration in older adults with DS, we would have used a test battery more closely resembling the one used in the MEADOWS study [17]. Conversely, if our target population had been younger than in the present study, we might have had to further adapt the test battery, given a recent report [41] showing that direct measures of short-term memory “had problematic floor effects, moderate test–retest reliability, some practice effects, and minimal convergent validity” when studied in participants with DS who were significantly younger (6–19 years old; $\bar{x}$ = 12.76; s = 3.22) than our studies’ samples.

As a practical consequence of the small number of properly powered, randomized clinical trials published in the field of DS to date, and the different goals of different trials, no single neuropsychological test battery can or should be considered as the gold standard in the field. For example, the test battery used in the pilot memantine study had the same historical origins as the ACTB; i.e., it was based in the work carried out by Drs. Bruce Pennington and Lynn Nadel research teams [4]. Analysis of the results of the pilot memantine study led us to adapt the battery by dropping one measure (the RBMT) and including a couple new ones (CANTAB SSP and a Go/No-go test) in the design of the follow-up memantine trial. Additionally, the primary measure for the new trial became the CVLT-II-sf, because it was the only test in which we were able to detect a significant drug effect.

Lessons learned from analyses contained in the present study will allow for the design of progressively refined, goal-specific test batteries. We have learned, for example, that although most psychometric properties of the CVLT-II-sf are adequate for inclusion in future test batteries, its test–retest reliability was less than ideal. For the CVLT-II-sf Total score, we observed a 22% increase in mean score from T1 to T2 and moderate ICC (0.67), whereas for the CVLT-II-sf Discriminability score, the T2 to T1 mean score difference was 43% higher than the baseline score and again the ICC was moderate (0.66). This practice effect is most likely a result of the repeated use of the standard test form (i.e., the same word list) in T1 and T2. A similar practice effect for the CVLT has been reported by others [42], who observed that the use of the alternative form of this test, consisting of a different word list, can effectively eliminate this issue. The use of a different word-list test, containing multiple lists of words, such as the one available in the NIH Toolbox could also prevent this drawback of the CVLT. Given our experience and all the data we have collected from adolescents and adults with DS using both the English and Portuguese versions of the CVLT-II-sf, however, our group is likely to continue using this instrument in future studies, but we plan to incorporate the multiple word lists contained in the 3rd version of the CVLT.

There were a few discrepancies between the initial exploratory analysis in which mean test scores across test sites were compared with either ANOVA (three sites) or *t*-tests (two sites) and the multiple regression analysis shown toward the end of Results. However, from the inspection of the values of the mean scores on these neuropsychological tests across sites, even when statistically significant differences were detected between sites, they were generally small and unlikely to be clinically relevant. It is also clear that type I errors were not an issue in the exploratory analysis, given that multiple regression analysis detected more statistically significant differences across sites than the ANOVA and *t*-tests. Perhaps the most noteworthy difference between these two approaches was the finding of significant site-location effects for the CVLT-II-sf Total and Free-Recall Discriminability scores through multiple regression analysis, which was not detected through ANOVA. Given that this neuropsychological instrument was the most sensitive to translation issues, it is possible that the multiple regression analysis may be revealing different valences of the translated nouns in Portuguese vs. English version, despite our best effort to generate culturally equivalent lists. Obviously, this specific issue is complex and may involve other individual and environmental differences between the groups, which would require the design of a specific study well beyond the scope of the present work. It should be noted that, for the purposes of the phase 2, follow-up memantine clinical trial [16], this issue was not critical, because in that study a mixed model was used to calculate whether the Mean Differences (T2−T1) between pairs of participants in placebo and memantine arms from the same site were different from each other (i.e., we calculated the differences between the differences in scores within groups). The mixed model we used in that study also took into account age, sex, site location, and SES.

Although the ICC for the SIB-R Broad Independence score was in the good range (0.75), the observed increase in score from T1 to T2 in the placebo arm of the study was 14% (Cohen “d” = 0.231), which likely reflected parent/caregiver expectations for potential treatment effects. An arguably more interesting observation, however, was that the SIB-R’s Maladaptive Index scores frequency histograms were best fitted with a negative exponential function (Figure A3), which showed a significant shorter time constant (τ) (i.e., shorter distribution tail) for São Paulo (τ = 2.9 [95% CI 2.4 to 3.6]) compared to either Cleveland (τ = 10.5 [95% CI 7.0 to 18.0]) or Denver (τ = 8.4 [95% CI 5.8 to 12.8]). This indicated a lower frequency of maladaptive behaviors in the Brazilian site sample in relation to the two US sites and was the only hint of a potential cultural/environmental factor significantly affecting cognitive test results between these countries. Presently, the primary shortcoming of the SIB-R is that the questionnaire has not been updated in a few decades. Consequently, it now contains several outdated questions (e.g., the participant’s ability to use a payphone), which makes it understandable that other adaptive-skills instruments (such as the Vineland Adaptive Behavior Scales) have been gaining broader adoption in the fields of DS and ID in general.

The SSP, Go-No-Go, and SWM have shown some of the least compelling psychometric properties among the measures comprising the trial battery, including poor correlation with other core measures. In light of these findings, plus a combined administration duration of approximately 35 min (SWM, 20 min; SSP, 5 min; Go-No-Go, 10 min) [16], it is unlikely that we will include any of these measures in future clinical trials in persons with DS.

Principal components and hierarchical clustering analyses of test results across 14 different measures identified two major ‘cognitive clusters’. This information may help us understand functional segregation structures that further validate the choice of short-term/working and episodic memory assessment instruments in the context of clinical trials with participants with DS. For example, it has been shown that short-term/working memory and general intelligence are interrelated and that both constructs correlate with academic and employment outcomes in those with and without DS [41,43,44,45]. One well-defined Euclidian proximity cluster comprised a measure of verbal short-term memory (DAS-II Digits Forward), two proxy measures of verbal intelligence (PPVT-4 and TROG-2), and one measure of adaptive skills (SIB-R Broad Independence score). A second cluster of interest contained two CVLT-II-sf sub-measures of episodic memory, one measure of long-term visual recognition memory (PRM), and one measure of non-verbal intelligence (DAS-II Matrices).

Finally, our analysis of CVLT-II-sf serial-position effects has confirmed recent findings of primacy/recency effects in individuals with DS performing list memory tasks [41]. These results are also in agreement with work published several decades ago showing that children with learning disability displayed both primacy and recency effects during the recall task of Atkinson, Hansen, and Bernback [46]. Our results also agree with previous work showing that the curvature of the U-shaped serial position curves diminishes across the five list acquisition trials of the Auditory Verbal Learning Test (AVLT) in neurotypical adults [34].

Although significant improvements have been made in the care of those with DS over the past three decades, current statistics on their cognitive development, schooling, employment, and life expectancy are not significantly different from what was reported 20 years ago [2]. Therefore, the development of an even moderately successful pharmacotherapy for the neurodevelopmental and/or neurodegenerative components of DS could have a significant impact on the quality of life of individuals affected by this disorder and their families and communities. The continued development and refinement of neuropsychological test batteries as well as reliable biomarkers of cognitive function and DSAD [13,47,48] are two critical steps to achieve this goal.

## Figures and Tables

**Table 1:** Task performance comparisons between sites at baseline and test score distribution properties. When test results were available for the three sites, an ANOVA was performed; otherwise a t test with Welsh correction was applied to compare the mean test results. Means and standard deviations derived from fitted unimodal Gaussians, 95% confidence intervals for these parameters, R^2^ values, and Shapiro–Wilk normality test results are also presented. The symbols “*” and “**” indicate “*p*” values < 0.05 and <0.01, respectively.

Measure	Site Comparisons (3 Sites ANOVA; 2 Sites *t*-Test)	Sample Mean Value $\bar{x}\;(95\%\mathrm{CI})$	Standard Deviation s (95% CI)	Goodness of Fit R^2^ Value	Shapiro–Wilk W; “*p*” Value
CVLT II Total Correct (1)	F(2, 181) = 1.2474; *p* = 0.2897	14.99 (12.67 to 17.15)	7.89 (6.07 to 10.75	0.6906	0.9599; 0.6596
CVLT-II Recall Discriminability (2)	F(2, 181) = 3.0259; *p* = 0.051	1.26 (1.04 to 1.49)	0.97 (0.76 to 1.27)	0.7577	0.9765; 0.8531
DAS-II Digits Forward Total (3)	F(2, 177) = 1.7044; *p* = 0.1849	9.25 (8.41 to 10.09)	4.36 (3.65 to 5.24)	0.9243	0.9717; 0.9280
PRM Total Correct (4)	F(2, 186) = 0.536; *p* = 0.5862	23.45 (21.11 to 25.85)	7.70 (5.88 to 11.13)	0.6577	0.8922; 0.0871
PAL 1st Memory Score (5)	F(2, 186) = 0.669; *p* = 0.5132	8.02 (7.26 to 8.71)	4.63 (3.91 to 5.63)	0.8449	0.9705; 0.8275
SWM Strategy (6)	F(2, 187) = 1.59; *p* = 0.2065	39.80 (39.71 to 39.89)	2.67 (2.57 to 2.77)	0.9979	0.9119; 0.1448
SWM Between Errors (7)	F(2, 187) = 2.377; *p* = 0.0957	68.29 (66.73 to 69.83)	10.48 (8.85 to 12.43)	0.9701	0.9391; 0.4870
SSP Span Length (8)	*p* = 0.5487 (Cleveland *n* = 53; São Paulo *n* = 71)	3.49 (2.74 to 4.11)	1.04 (0 to 2.21)	0.9529	0.8678; 0.2578
SSP Usage Errors (9)	*p* = 0.0395 * (Cleveland *n* = 53; São Paulo *n* = 71)	2.58 (1.11 to 3.04)	1.63 (1.24 to 2.26)	0.9055	0.9672; 0.8698
		1.73 (0.59 to 2.94)	1.64 (1.14 to 2.94)	0.9055	0.9672; 0.8698
Go-No-Go Response Time (ms) (10)	*p* = 0.0082 ** (Cleveland *n* = 53; São Paulo *n* = 71)	Cleveland: 481 (422 to 549)	118 (54.8 to 237)	0.6294	0.9369; 0.44589
		São Paulo: 546 (503 to 588)	140 (100 to 211)	0.7644	0.9578; 0.7517
DAS-II Matrices Ability Score (11)	F(2, 180) = 0.003; *p* = 0.9966	49.31 (47.65 to 50.86)	7.94 (6.67 to 9.49)	0.9231	0.9411; 0.3320
PPVT-4 Raw Score (12)	*p* = 0.2606 (t test; Cleveland vs. São Paulo)	112.6 (101.1 to 123.2)	40.86 (30.20 to 53.87)	0.8709	0.9205; 0.2898
TROG-2 Total Correct (13)	F(2, 187) = 6.501; *p* = 0.0019 **	18.43 (17.15 to 19.68)	6.65 (5.49 to 8.22)	0.8689	0.9756; 0.9306
SIB-R Broad Independence (14)	F(2, 185) = 3.058; *p* = 0.049 * (no post hoc differences)	44.10 (37.68 to 50.23)	26.14 (20.38 to 33.77)	0.8472	0.8926; 0.1059

**Table 2:** Test–retest reliability was assessed by comparing baseline (T1) and post-treatment (T2) aggregated test scores from the placebo arms at the three clinical trial sites. This table shows mean differences between test scores, Cohen’s d’s, and intra-class correlations (ICCs). Cohen’s d’s interpretation: <0.2 = small effect size and 0.2 to 0.6 = medium effect size (denoted here by the symbol “+”). ICC interpretation: <0.5 = poor (symbolized in the table by a “†”); 0.5 to 0.74 = moderate; 0.75 to 0.90 = good; >0.90 = excellent. (* Note that all ICCs were significantly greater than 0, at *p* < 0.001).

Measure	Placebo Baseline Scores $\bar{x}\pm \mathrm{s (n)}$	Placebo Retest Scores $\bar{x}\;\mathrm{(s)}\pm \mathrm{s (n)}$	Mean Differences $\bar{x}\;\mathrm{(s)}\pm \mathrm{s (n)}$	Cohen’s d	ICC *
CVLT II Total Correct (1)	14.18 ± 7.03 (95)	17.33 ± 7.06 (95)	3.15 ± 5.17 (95)	0.447 ^+^	0.67
CVLT-II Recall Discriminability (2)	0.96 ± 0.88 (95)	1.37 ± 0.81 (95)	0.41 ± 0.62 (95)	0.485 ^+^	0.66
DAS-II Digits Forward Total (3)	8.49 ± 4.46 (95)	8.12 ± 4.45 (95)	−0.07 ± 2.48 (95)	−0.083	0.85
PRM Total Correct (4)	21.40 ± 6.23 (94)	21.62 ± 6.31 (94)	0.22 ± 4.16 (94)	0.035	0.79
PAL 1st Memory Score (5)	7.68 ± 4.40 (94)	8.62 ± 4.73 (94)	0.91 ± 3.71 (94)	0.206	0.66
SWM Strategy (6)	38.49 ± 3.78 (95)	38.02 ± 3.83 (95)	−0.63 ± 3.64 (95)	−0.124	0.51
SWM Between Errors (7)	71.74 ± 16.78 (95)	73.16 ± 23.74 (95)	0.14 ± 12.82 (95)	0.069	0.83
SSP Span Length (8)	2.78 ± 1.77 (76)	2.91 ± 1.57 (76)	0.13 ± 1.30 (76)	0.078	0.70
SSP Usage Errors (9)	2.36 ± 1.85 (76)	2.20 ± 1.76 (76)	−0.16 ± 1.96 (76)	−0.089	0.41 ^†^
Go-No-Go Response Time (ms) (10)	557.1 ± 135.4 (72)	559.5 ± 147.1 (72)	2.40 ± 96.8 (72)	0.017	0.77
DAS-II Matrices Ability Score (11)	51.68 ± 12.51 (95)	52.13 ± 13.15 (95)	0.54 ± 8.89 (95)	0.035	0.75
PPVT-4 Raw Score (12)	109.12 ± 37.78 (76)	113.58 ± 38.73 (76)	4.46 ± 12.96 (76)	0.117	0.94
TROG-2 Total Correct (13)	18.75 ± 5.90 (95)	19.48 ± 7.49 (95)	0.31 ± 4.97 (95)	0.108	0.79
SIB-R Broad Independence (14)	44.30 ± 25.67 (91)	50.42 ± 27.29 (91)	6.12 ± 18.09 (91)	0.231 ^+^	0.75

**Figure A2:**
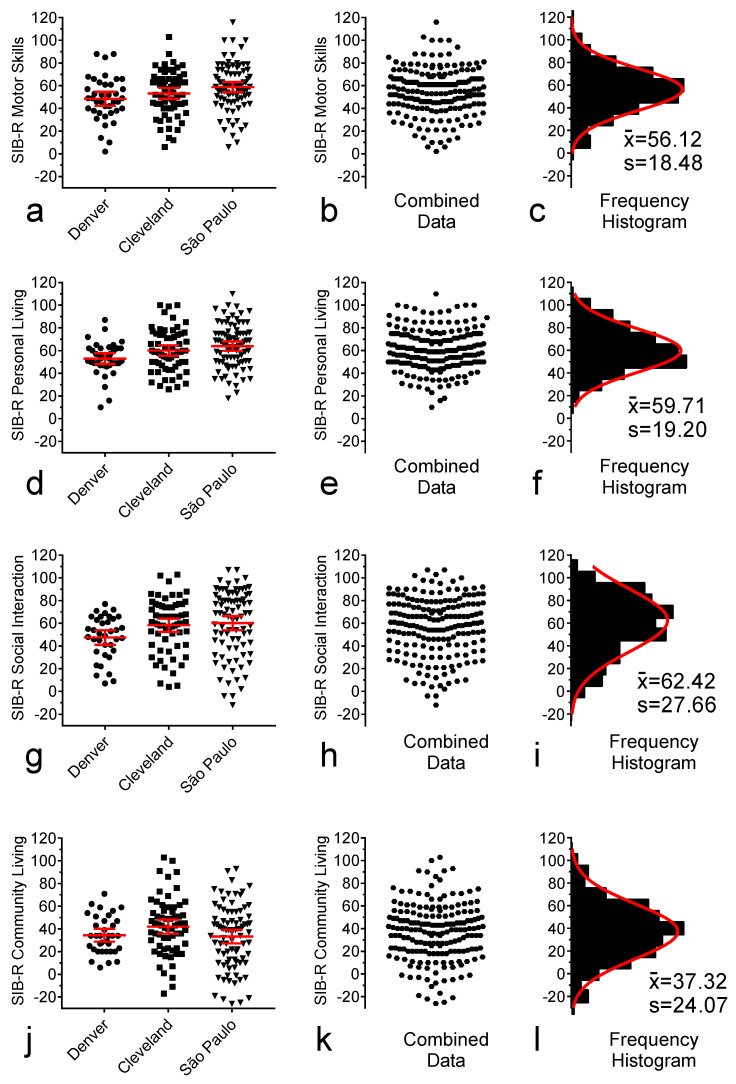
Distribution of individual baseline test scores for four SIB-R sub-measures: Motor Skills, Personal Living, Social Interaction, and Community Living assessed in Denver (●), Cleveland (■), and São Paulo (▼). (**a**,**d**,**g**,**j**) Scatter plots of test scores in each of the three sites. (**b**,**e**,**h**,**k**) Combined scores for each of these four measures at the three study sites. (**c**,**f**,**i**,**l**) Frequency histograms for combined scores and single Gaussian curve fittings of the combined data for each of the four SIB-R sub-measures in Denver, Cleveland, and São Paulo. Solid symbols represent computed individual scores and red lines in (**a**,**d**,**g**,**j**) represent mean ± 95% CI.

## Data Availability

The original contributions presented in this study are included in the article. Further inquiries can be directed to the corresponding author.

## Abbreviations

The following abbreviations are used in this manuscript:

DS	Down syndrome
AD	Alzheimer’s disease
DSAD	Alzheimer’s disease associated with Down syndrome
NMDA	N-Methyl-D-aspartate
ID	Intellectual Disability
CVLT-II	California Verbal Learning Test 2nd Edition
CVLT-II-sf	California Verbal Learning Test 2nd Edition short form
CVLT-III	California Verbal Learning Test 3rd Edition
DAS-II	Differential Ability Scales-Second Edition
CANTAB	Cambridge Neuropsychological Test Automated Battery
PRM	Pattern Recognition Memory
PAL	Paired Associates Learning
RBMT	Rivermead Behavioral Memory Test
SWM	Spatial Working Memory
SSP	Spatial Span
PPVT-III	Peabody Picture Vocabulary Test-3rd edition
PPVT-IV	Peabody Picture Vocabulary Test-4th edition
TROG-II	Test of Reception of Grammar-2nd edition
SIB-R	Scales of Independent Behavior–Revised
ANOVA	Analysis of Variance
PCA	Principal Component Analysis
ICC	Intra-class Correlation
CI	Confidence Interval
AVLT	Auditory Verbal Learning Test

## Appendix A

Inclusion and Exclusion Criteria (these applied to both the Pilot and Follow-up Memantine Trials): participants who fulfilled all the inclusion criteria and none of the exclusion criteria listed below were accepted for enrollment. The benefits and risks of participation in the studies were explained to all subjects and respective caregivers prior to screening for the studies. At that time, written informed consent was obtained from all parties prior to initiating formal screening for the studies.
  Inclusion Criteria: Cytogenetically documented trisomy 21 or complete unbalanced translocation of Chromosome 21. Mosaic DS and partial translocations were excluded from the study. No pregnancy by serum testing at screening. Females of child-bearing potential, sexually active had to be practicing a reliable method of birth control. Pregnancy tests were performed at all follow-up medical visits. Laboratory findings within normal limits or judged clinically insignificant at baseline. Vital signs within normal limits for age (stable, medically treated hypotension was allowed). Electrocardiogram (ECG) had to demonstrate predominately normal sinus rhythm. (Minor abnormalities documented as clinically insignificant were allowed.) Participants who have received any experimental drug for DS had to undergo a washout. All participants had to be in general good health; be able to swallow oral medication; have a reliable caregiver or family member who agrees to accompany the participant to all visits, provides information about the participant as required by the protocol, and ensures compliance; and be sufficiently proficient in English to reliably complete the study assessments.
  Exclusion Criteria: Participant weighing less than 40 kg. Current psychiatric or neurologic diagnosis other than DS (e.g., schizophrenia, bipolar disorder, clinical dementia). Current treatment with psychotropic drugs. Drug or alcohol abuse or dependence. Significant suicide risk or who would require treatment with electro-convulsive therapy or with psychotropic drugs during the study or who have received treatment with a depot neuroleptic drug within 6 months of entering the study. Current or expected hospitalization or residence in a skilled nursing facility (could reside in group homes or other residential settings with no skilled nursing). Active or clinically significant conditions affecting absorption, distribution, or metabolism of study drug (e.g., inflammatory bowel disease, celiac disease, or renal insufficiency). Significant allergies to or other significant intolerance of or with contraindications to memantine therapy, or their ingredients, as stated in the prescribing information. Expected to require general anesthetics during the course of the study. Presence or recent history of seizure disorder (<3 years). Clinically significant and/or clinically unstable systemic disease. [Those with controlled hypothyroidism had to be on a stable dose of medication for at least 3 months prior to screening and have normal serum thyroid-stimulating hormone (TSH) levels at screening; and those with controlled diabetes mellitus had to have a hemoglobin A1C (HbA1c) reading of <8.0% and a random serum glucose value of <170 mg/dL.] Severe infections or a major surgical operation within 3 months prior to screening. History of persistent cognitive deficits immediately following head trauma. Donation of blood or blood products in less than 30 days prior to screening or while participating in the study. Inability to comply with the protocol or perform the outcomes measures due to hearing or visual impairment or other clinically relevant issues.

## Appendix B

CVLT-II-sf Word List, Portuguese Translation: Several research team members worked in making sure that we would get the closest possible correspondence between the English and Portuguese versions of the CVLT. All the team members involved in generating the Portuguese word list are reasonably fluent in both English and Portuguese.

Best efforts were made not only to translate the words in the lists, but, in a few instances, to substitute the original words in the English list with Portuguese words in the same category that had comparable syntax, number of syllables, and potentially a similar level of familiarity to the study participants in both countries. Table A1 contains the original CVLT-II-sf English word list and its Portuguese counterpart.

**Table A1:** Original CVLT-II-sf English word list and its Portuguese counterpart. A key hurdle we encountered in designing the phase 2, follow-up memantine study was that the CVLT-II sf was neither translated nor validated in Portuguese, which led us to produce our own Portuguese adaptation of the word list. In this process, we realized early on that simply directly translating word for word was not the best approach. Cultural factors related to the familiarity of the word had to be considered. For example, ‘cherries’ translate to ‘cerejas’ in Portuguese, however, ‘cherries’ are not commonly consumed in most parts of Brazil, which is the reason we instead chose the word ‘uvas’, which is the Portuguese word for ‘grapes’. Other words, such as ‘wrench’, whose direct Portuguese translation is ‘chave inglesa’, is both a compound word in Portugues and the tool itself is not as popular in Brazil as the ‘hammer’ (‘martelo’), which was the corresponding word for the tool that we replaced it with in the list. The same was true for the tool ‘drill’, which we replaced with the Portuguese word ‘serrote’, which means ‘hacksaw’. Here, however, we found that participants, instead of recalling the word ‘serrote’, would occasionally recall the word ‘serra’ (which translates to ‘saw’ in English but also means ‘mountain chain’ in Portuguese; and hence why we originally decided to choose ‘serrote’ instead of ‘serra’). Because of the possibility of confusion, this particular word may need to be revised in future applications of the CVLT-II sf list in Portuguese. A final example of an apparently unconventional Portuguese equivalent choice was not to use the word ‘suéter’, which is the lusitanized version of the English word ‘sweater’ and the precise translation. However, due to the tropical climate of most of Brazil, this clothing item is rarely used in most of the country, which is the reason it was replaced with the word ‘blusa’ (‘blouse’ or ‘shirt’ in English).

Word List (English)	Word List (Portuguese)
hat	chapéu
cherries	uvas
wrench	martelo
sweater	blusa
lemon	limão
pliers	alicate
belt	cinto
peaches	peras
drill	serrote
Long-term Recognition Intrusion Lists (Participants are asked whether they have heard the following words)	
List 1 (English)	List 1 (Portuguese)
newspaper	jornal
wrench	martelo
apple	maçã
screwdriver	parafuso
peaches	peras
shirt	camisa
typewriter	computador
coffee	café
sweater	blusa
List 2 (English)	List 2 (Portuguese)
cherries	uvas
pants	calça
vitamins	vitamina
kite	pipa
hammer	furadeira
spoon	colher
banana	banana
drill	serrote
hat	chapéu
List 3 (English)	List 3 (Portuguese)
elbow	cotovelo
socks	meia
daisy	rosa
pliers	alicate
orange	laranja
belt	cinto
saw	prego
lemon	limão
gasoline	gasolina

## Appendix C

Additional Data Analysis

**Table A2:** Additional descriptive statistics for task performance scores at baseline. For each measure, the number participants minus the number of participants at floor was calculated and the percentage of participants at floor was displayed. Arithmetic means and standard deviations, as well as medians, minimum, low quartile, upper quartile, and maximum values for each measure at baseline were also calculated as a first approximation of the score’s distributions. Skew and kurtosis values were listed to provide further quantitative description of the distribution histogram properties for the scores for each measure. Distribution skewness interpretation: 0–0.5, approximately symmetric; 0.5 to 1 moderately skewed (denoted in the table by the symbol “+”); >1 highly skewed (represented by “++”). Kurtosis (*excess kurtosis*) interpretation: for the purposes of this analysis, −1 to 1 was considered approximately mesokurtic, whereas > 1 indicated a leptokurtic distribution, i.e., data distribution with heavy tails and higher likelihood of outliers, which was symbolized in the table by a “†”.

Measure	Number Participants Minus Participants at Floor (% Participants at Floor)	Baseline Scores Arithmetic Mean (Standard Deviation)	Median (Min; L-Qtr; U-Qrt; Max)	Skew	Kurtosis
CVLT II Total Correct (1)	190 − 5 = 185 (2.63%)	14.12 (6.57)	15.00 (1.00; 9.00; 20.00; 29.00)	−0.112	−0.947
CVLT-II Recall Discriminability (2)	190 − 5 = 185 (2.63%)	1.03 (0.83)	1.10 (−1.5; 0.30; 1.70; 2.60)	−0.401	0.543
DAS-II Digits Forward Total (3)	190 − 10 = 180 (5.26%)	8.96 (3.98)	9 (1; 6; 11.50; 22)	0.337	−0.057
PRM Total Correct (4)	190 − 0 (0%)	22.78 (6.14)	23 (10; 18; 28; 36)	−0.015	−0.955
PAL 1st Memory Score (5)	190 − 12 = 178 (6.32%)	8.19 (3.90)	8 (1; 5; 11; 17)	0.059	−0.689
SWM Strategy (6)	190 − 0 (0%)	38.63 (3.55)	39 (19; 37; 41; 47)	−1.378 ^++^	5.052 ^†^
SWM Between Errors (7)	190 − 0 (0%)	70.83 (17.85)	68 (16; 61; 79; 176)	1.419 ^++^	6.528 ^†^
SSP Span Length (8)	150 − 26 = 124 (17.33%)	3.59 (0.95)	3.5 (2; 3; 4; 6)	0.227	−0.533
SSP Usage Errors (9)	150 − 26 = 124 (17.33%)	2.49 (1.77)	2 (0; 1; 3; 8)	0.799^+^	0.575
Go-No-Go Response Time (ms) (10)	147 − 0 = 147 (0%)	557(131)	528 (326; 452; 646; 885)	0.576 ^+^	−0.499
DAS-II Matrices Ability Score (11)	185 − 6 = 179 (3.24%)	52.33 (11.06)	52 (18; 47; 57; 100)	0.373	2.820 ^†^
PPVT-4 Raw Score (12)	150 − 0 = 150 (0%)	108 (39.34)	113 (8; 80; 135; 205)	−0.090	−0.467
TROG-2 Total Correct (13)	150 − 0 (0%)	18.67 (5.83)	18 (8; 14; 23; 35)	0.361	−0.405
SIB-R Broad Independence (14)	189 − 0 (0%)	41.53 (24.67)	43 (−14; 25; 59; 98)	−0.157	−0.489

**Table A3:** Pearson’s r correlations between the different measures comprising the test battery. Asterisks indicate “*p*” values < 0.05, and values in boldface indicate “r” values ≥ 0.5.

	Pearson’s r Correlations Between Different Measures												
Measure	(2)	(3)	(4)	(5)	(6)	(7)	(8)	(9)	(10)	(11)	(12)	(13)	(14)
CVLT Total (1)	**0.85 ***	0.48 *	0.33 *	0.21 *	0.05	−0.34 *	0.41 *	−0.04	−0.09	0.37 *	**0.50 ***	0.34 *	0.29 *
CVLT Discrim. (2)		**0.50 ***	0.40 *	0.30 *	0.02	−0.37 *	0.42 *	−0.12	−0.05	0.40 *	**0.58 ***	0.47 *	0.38 *
DAS-II Digits (3)			0.36 *	0.20 *	−0.16	**−0.53 ***	**0.53 ***	0.09	−0.19 *	0.40 *	**0.61 ***	**0.59 ***	0.43 *
PRM Total (4)				0.35 *	−0.03	−0.26 *	0.40 *	0.04	−0.15	0.37 *	**0.53 ***	**0.52 ***	0.33 *
PAL First (5)					0.13	−0.22 *	0.28 *	−0.14	−0.02	0.24 *	0.30 *	0.24 *	0.17
SMW Strategy (6)						0.29 *	−0.05	0.10	−0.16	−0.13	−0.02	−0.11	−0.13
SMW Betw. Errors (7)							**−0.53 ***	0.07	0.14	**−0.54 ***	−0.46 *	−0.46 *	−0.45 *
SSP Span Length (8)								0.16	−0.34 *	0.33 *	**0.62 ***	0.48 *	0.48 *
SSP Errors (9)									−0.17	−0.13	0.01	0.01	−0.08
Go-No-Go Speed (10)										−0.01	−0.20 *	−0.12	−0.017
DAS-II Matrices (11)											0.46 *	0.39 *	0.32 *
PPVT-IV (12)												**0.73 ***	**0.50 ***
TROG (13)													0.43 *
SIB-R Broad Independ. (14)													

**Table A4:** Set of all Euclidean distances between the different measures comprising the test battery calculated from the three principal components obtained from the PCA graph depicted in Figure 4a. Asterisks indicate “*p*” values < 0.05, and values in boldface indicate “r” values ≥ 0.5.

	Euclidian Distances Between Different Measures												
Measure	(2)	(3)	(4)	(5)	(6)	(7)	(8)	(9)	(10)	(11)	(12)	(13)	(14)
CVLT Total (1)	**0.85 ***	0.48 *	0.33 *	0.21 *	0.05	−0.34 *	0.41 *	−0.04	−0.09	0.37 *	**0.50 ***	0.34 *	0.29 *
CVLT Discrim. (2)		**0.50 ***	0.40 *	0.30 *	0.02	−0.37 *	0.42 *	−0.12	−0.05	0.40 *	**0.58 ***	0.47 *	0.38 *
DAS-II Digits (3)			0.36 *	0.20 *	−0.16	**−0.53 ***	**0.53 ***	0.09	−0.19 *	0.40 *	**0.61 ***	**0.59 ***	0.43 *
PRM Total (4)				0.35 *	−0.03	−0.26 *	0.40 *	0.04	−0.15	0.37 *	**0.53 ***	**0.52 ***	0.33 *
PAL First (5)					0.13	−0.22 *	0.28 *	−0.14	−0.02	0.24 *	0.30 *	0.24 *	0.17
SMW Strategy (6)						0.29 *	−0.05	0.10	−0.16	−0.13	−0.02	−0.11	−0.13
SMW Betw. Errors (7)							**−0.53 ***	0.07	0.14	**−0.54 ***	−0.46 *	−0.46 *	−0.45 *
SSP Span Length (8)								0.16	−0.34 *	0.33 *	**0.62 ***	0.48 *	0.48 *
SSP Errors (9)									−0.17	−0.13	0.01	0.01	−0.08
Go-No-Go Speed (10)										−0.01	−0.20 *	−0.12	−0.017
DAS-II Matrices (11)											0.46 *	0.39 *	0.32 *
PPVT-IV (12)												**0.73 ***	**0.50 ***
TROG (13)													0.43 *
SIB-R Broad Independ. (14)													
